# Notoginsenoside R1 attenuates oxidative stress‐induced osteoblast dysfunction through JNK signalling pathway

**DOI:** 10.1111/jcmm.17054

**Published:** 2021-11-16

**Authors:** Xumin Li, Haiyan Lin, Xiaorong Zhang, Richard T. Jaspers, Qihao Yu, Yinghui Ji, Tim Forouzanfar, Dongyun Wang, Shengbin Huang, Gang Wu

**Affiliations:** ^1^ Department of Prosthodontics School and Hospital of Stomatology Wenzhou Medical University Wenzhou PR China; ^2^ Institute of Stomatology School and Hospital of Stomatology Wenzhou Medical University Wenzhou PR China; ^3^ Department of Oral and Maxillofacial Surgery/Pathology Amsterdam UMC and Academic Centre for Dentistry Amsterdam (ACTA) Vrije Universiteit Amsterdam (VUA) Amsterdam Movement Science Amsterdam The Netherlands; ^4^ Laboratory for Myology Amsterdam Movement Sciences Faculty of Behavioral and Movement Sciences Vrije Universiteit Amsterdam (VUA) Amsterdam The Netherlands; ^5^ Savaid Stomatology School Hangzhou Medical College Hangzhou PR China; ^6^ Department of Endodontics School and Hospital of Stomatology Wenzhou Medical University Wenzhou PR China; ^7^ Stomatological Center Peking University Shenzhen Hospital Shenzhen PR China; ^8^ Department of Oral Cell Biology Academic Centre for Dentistry Amsterdam (ACTA) University of Amsterdam (UvA) and Vrije Universiteit Amsterdam (VU) Amsterdam The Netherlands

**Keywords:** dysfunction, JNK, mitochondria, NGR1, osteoblast, oxidative stress

## Abstract

Oxidative stress (OS)‐induced mitochondrial damage and the subsequent osteoblast dysfunction contributes to the initiation and progression of osteoporosis. Notoginsenoside R1 (NGR1), isolated from *Panax notoginseng*, has potent antioxidant effects and has been widely used in traditional Chinese medicine. This study aimed to investigate the protective property and mechanism of NGR1 on oxidative‐damaged osteoblast. Osteoblastic MC3T3‐E1 cells were pretreated with NGR1 24 h before hydrogen peroxide administration simulating OS attack. Cell viability, apoptosis rate, osteogenic activity and markers of mitochondrial function were examined. The role of C‐Jun N‐terminal kinase (JNK) signalling pathway on oxidative injured osteoblast and mitochondrial function was also detected. Our data indicate that NGR1 (25 μM) could reduce apoptosis as well as restore osteoblast viability and osteogenic differentiation. NGR1 also reduced OS‐induced mitochondrial ROS and restored mitochondrial membrane potential, adenosine triphosphate production and mitochondrial DNA copy number. NGR1 could block JNK pathway and antagonize the destructive effects of OS. JNK inhibitor (SP600125) mimicked the protective effects of NGR1while JNK agonist (Anisomycin) abolished it. These data indicated that NGR1 could significantly attenuate OS‐induced mitochondrial damage and restore osteogenic differentiation of osteoblast via suppressing JNK signalling pathway activation, thus becoming a promising agent in treating osteoporosis.

## 1 INTRODUCTION

Osteoporosis is defined as a systemic degenerative disease, which is characterized by dysregulation of bone formation and progressive bone micro‐architectural deterioration.^1^ Osteoporosis largely increases the fracture risk of bone.^1^, ^2^ One of the major pathogenic factors for osteoporosis is oxidative stress (OS)^3^, ^4^—a pathophysiological status with relatively overproduced reactive oxygen species (ROS) and insufficient anti‐oxidative defence.^5^ Our meta‐analysis of clinical data concludes that in postmenopausal women the status of OS is closely related to the decreased bone mineral density (BMD).^6^

Osteoblasts are responsible for bone formation, playing a crucial role in maintaining BMD and bone microstructure.^7^ OS can significantly reduce osteoblast viability and activity, thereby diminishing osteoblast quantity and function,^8^ which leads to the onset and progression of osteoporosis.^9^ At subcellular level, ROS directly attack and cause damage to mitochondria—the most vulnerable target of ROS, which stimulates mitochondria to further generate and release ROS, forming a vicious circle and finally leading to mitochondrial dysfunction.^10^ Mitochondrial dysfunction forms a critical molecular mechanism accounting for the OS‐induced osteoblast apoptosis.^11^ Mitochondrial dysfunction also further impairs osteoblastic bone formation function.^12^ Therefore, bioactive agents that can both relieve OS‐induced mitochondrial dysfunction and restore osteoblast function are promising to treat osteoporosis.

One of such candidate bioactive agents is notoginsenoside R1 (NGR1), a natural triterpene saponin compound derived from the traditional Chinese herb *Panax notoginseng*.^13^ Firstly, NGR1 has a potent capacity in relieving cellular damages, thereby having a strong protective effect on different kinds of cells,^14^, ^15^, ^16^ from OS in several pathological situations. Furthermore, the anti‐oxidative effect of NGR1 is largely attributed to its preventive effect on OS‐induced mitochondrial dysfunction.^17^, ^18^ On the other hand, NGR1 also has an invaluable property to promote osteoblast function—osteogenic differentiation.^19^, ^20^ However, it remains to be elucidated whether NGR1 can prevent OS‐induced mitochondrial dysfunction and restore osteoblast function.

C‐Jun N‐terminal kinase (JNK) is one of the three signalling pathways of mitogen‐activated protein kinases (MAPKs) that mediate cellular responses to physiological and pathological stimuli.^21^ JNK pathway can be activated in response to ROS and mediates OS‐induced mitochondrial dysfunction in primary cortical neurons.^22^ Furthermore, JNK activation by acetaminophen is also shown to inhibit mitochondrial bioenergetics^23^ and mitochondrial biogenesis^24^ in liver cells. Consistently, our preliminary experiment also showed that JNK signalling pathway is activated in osteoblast under OS stimulation. On the other hand, NGR1 has been shown to be capable of blocking JNK signalling pathway.^25^ Therefore, we hypothesized that NGR1 relieves OS‐induced osteoblast dysfunction and mitochondrial damage through attenuating OS‐induced JNK activation.

In the present study, we established an OS‐induced osteoblast dysfunction model to explore (1) NGR1’s osteoblast protection against oxidative damage and (2) its underlying molecular mechanisms.

## 2 MATERIALS AND METHODS

### 2.1 Cell culture and treatment

The mouse pre‐osteoblast MC3T3‐E1 cells were purchased from ATCC and cultured in α‐minimum essential medium (α‐MEM; Gibco) with 10% foetal bovine serum (FBS; Gibco), 100 units/ml penicillin (Invitrogen) and 100 mg/ml streptomycin (Invitrogen) in a humidified atmosphere of 5% CO_2_ at 37°C. MC3T3‐E1 cells were pretreated with 0–50 μmol/L NGR1 (Zelang) diluted by dimethyl sulphoxide (DMSO; Sigma) for 24 h. The final concentration of DMSO in all experiments was <0.5%. The other compounds’ treating conditions were as follows: Hydrogen peroxide (H_2_O_2_) (0.75 mM, Sigma) for 6 h; SP600125 (100 μmol/L; Sigma), Anisomycin (100 μmol/L; Sigma) pre‐incubated for 1 h. Osteogenic medium (OM) used for osteogenic inducing contains basic medium, β‐glycerol phosphate (10 mM; Sigma) and ascorbic acid (50 μg/ml; Sigma).

### 2.2 Cell viability

1 × 10^4^ MC3T3‐E1 cells were seeded per well in 96‐well plates and exposed to H_2_O_2_ and/or indicated test compounds. 3(4,5‐dimethylthiazol‐2‐yl)‐2,5‐diphenyltetrazolium (MTT) colorimetric assay was used for cell viability examination as previously described.^8^

### 2.3 Measurement of apoptosis by transferase dUTP nick end labelling (TUNEL) assay

3 × 10^4^ MC3T3‐E1 cells were seeded per well in 48‐well plates with coverslip. TUNEL staining was processed as previously described.^8^ The percentage of apoptotic cells was estimated by the TUNEL positive cell counts in total cells from random fields.

### 2.4 Alkaline phosphatase (ALP) staining and ALP activity assay

3 × 10^4^ MC3T3‐E1 cells per well were seeded on 48‐well plates and exposed to H_2_O_2_ and/or other test compounds for indicated time. Then, the medium was exchanged to OM for 7 days. ALP staining was performed as previously described.^8^ Each well was photographed using a stereomicroscope (Olympus). ALP activity was assayed as previously described^8^ and presented as the concentration per gram of protein ((mg/ml)/g protein). Protein concentration was determined using BCA protein assay (Thermo Fisher).

### 2.5 Mineralization analysis

Mineralization of MC3T3‐E1 cells was determined in 48‐well plates using Alizarin red S staining (ARS) as previously described.^8^ Each well was photographed under stereomicroscope, and the mineralization area was quantified by Image J.

### 2.6 Real‐time polymerase chain reaction (rt‐PCR)

30 × 10^4^ MC3T3‐E1 cells per well were seeded on 6‐well plates and exposed to H_2_O_2_ and/or other test compounds for indicated time. Then, the medium was exchanged to OM for 7 days. Total RNA extraction and rt‐PCR amplifications were performed as previously described.^8^ The sequences of specific primers were listed in Table 1.

**TABLE 1:** Primers sequences for polymerase chain reaction (PCR)

Gene	Forward primer (5’−3’)	Reverse primer (5’−3’)
Akp2 (ALP)	TGCCTACTTGTGTGGCGTGAA	TCACCCGAGTGGTAGTCACAATG
Osteocalcin (OCN)	AGCAGCTTGGCCCAGACCTA	TAGCGCCGGAGTCTGTTCACTAC
Collagen I (COL I)	ATGCCGCGACCTCAAGATG	TGAGGCACAGACGGCTGAGTA
Runt‐related transcription factor 2 (Runx2)	CACTGGCGGTGCAACAAGA	TTTCATAACAGCGGAGGCATTTC
Glyceraldehyde−3‐phosphate dehydrogenase (GAPDH)	TCAACAGCAACTCCCACTCTT	ACCCTGTTGCTGTAGCCGTATTCA
cytochrome c oxidase subunit 1 (COX−1)	ATTGCCCTCCCCTCTCTACGCA	CGTAGCTTCAGTATCATTGGTGCCC
β‐actin	CCATGTTCCAAAACCATTCC	GGGCAACCTTCCCAATAAAT

### 2.7 Measurement of mitochondrial membrane potential (MMP)

To assess MMP, cells were co‐stained with tetramethylrhodamine methyl ester (TMRM, 100 nM, Life Technologies) and Mitotracker Green (Mitogreen, 100 nM, Life Technologies) for 30 min, as in our previous study.^11^ Images were captured under a fluorescence microscope (Leica DMIL). Excitation wavelengths were 543 nm for TMRM and 488 nm for Mitogreen. Post‐acquisition processing was performed with Image J software for the quantification of fluorescent intensity.

### 2.8 Measurement of mitochondrial superoxide production

Cellular superoxide production in mitochondria was detected using the MitoSOX™ Red mitochondrial superoxide indicator (Life Technologies) according to the manufacturer's protocol and a previously published method.^26^ Briefly, adherent MC3T3‐E1 cells from different groups were co‐incubated with MitoSOX™ reagent working solution (5 μM) and Mitogreen (100 nM) at 37°C in the dark for 10 min. After incubation, cells were rinsed 3 times using warm Hank's balanced salt solution containing Ca2+/Mg2+ (HBSS; Gibco). Images were captured under a fluorescence microscope (Leica DMIL). Excitation wavelengths were 543 nm for MitoSOX and 488 nm for Mitogreen. Post‐acquisition processing was performed with Image J software for the quantification and measurement of fluorescent intensity.

### 2.9 Detection of adenosine triphosphate (ATP) production

For the measurement of ATP level, whole‐cell extracts were lysed in lysis buffer provided in the ATP assay kit (Beyotime). After centrifugation at 12,000 g for 5 min at 4°C, the supernatants were transferred to a new 1.5 ml tube for ATP analysis. The luminescence from a 100 μL sample was assayed in a luminometer (Molecular Devices) together with 100 μL of ATP detection buffer. A standard curve of ATP concentrations (1 nM–1 µM) was prepared from a known amount.

### 2.10 Evaluation of mitochondrial DNA (MtDNA) copy number

MC3T3‐E1 cells were lysed for total DNA extraction. rt‐PCR was conducted with 40 ng DNA (OD260; NanoDrop). MtDNA copy number was measured by cytochrome c oxidase subunit 1 (COX‐1) normalized with nuclear DNA products (β‐actin). The sequences of specific primers were listed in Table 1.

### 2.11 Western blotting assay

For Western blotting assay, MC3T3‐E1 cells were lysed with RIPA lysis buffer (Sigma) supplemented with protease and phosphatase inhibitors (Thermo Fisher). The following steps were performed as previously described.^8^ Anti‐pJNK (1:2000, Cell Signaling) and anti‐JNK (1:2000, Cell Signaling) were the primary antibodies used in this assay. The secondary horseradish peroxidase‐conjugated anti‐rabbit IgG antibody (1:4000, Invitrogen) was incubated for 1 h. Each protein bands were quantified by Image J software, and the relative levels of pJNK to JNK were measured.

### 2.12 Statistical analyses

Each experiment was repeated in triplicate. Data were reported as mean ± standard deviation (SD). One‐way analysis of variance (ANOVA) was carried out by GraphPad Prism Software (Graph Pad Software). *p* values < 0.05 were recognized statistically significant.

## 3 RESULTS

### 3.1 NGR1 attenuated H_2_O_2_‐induced osteoblast apoptosis and dysfunction

**FIGURE 1:**
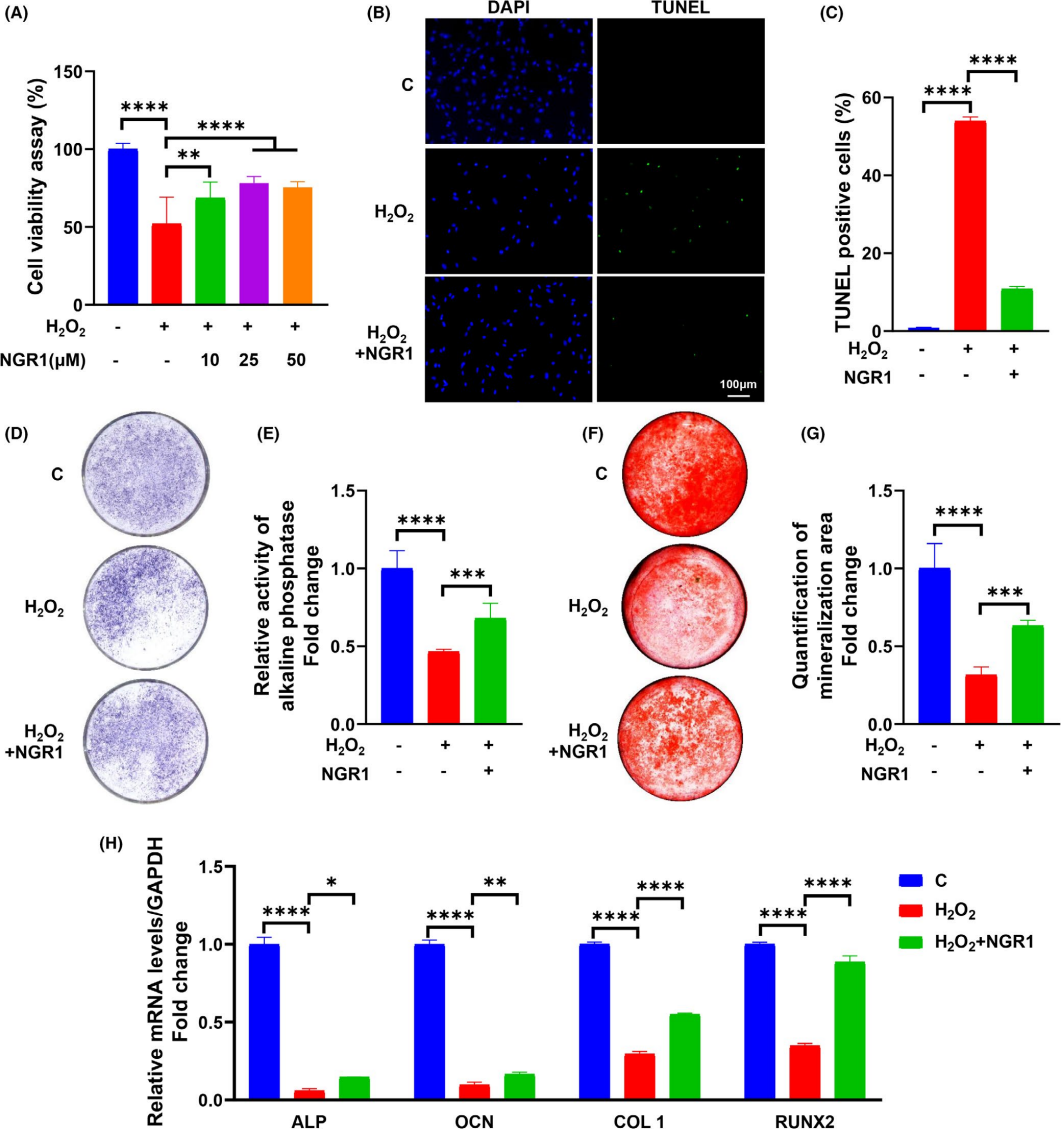
NGR1 attenuated H_2_O_2_‐induced osteoblast apoptosis and dysfunction. (A) Cell viability was determined by 3(4,5‐dimethylthiazol‐2‐yl)‐2,5‐diphenyltetrazolium (MTT) reduction of MC3T3‐E1 cells in the presence of 10, 25 or 50 μM NGR1 for 24 h before treating with 0.75 mM H_2_O_2_ for 6 h. (B, C) Cells were stained for Transferase dUTP Nick End Labeling (TUNEL) (green). 4’, 6‐diamidino‐2‐phenylindole (DAPI) was used to stain the nuclei. Scale bars, 100 μm. (D) MC3T3‐E1 cells after indicated treatment were subjected to alkaline phosphatase (ALP) staining and (E) ALP activity test. (F) Mineralization area of MC3T3‐E1 cells after osteogenic inducing for 4 weeks was determined by alizarin red S staining. (G) The quantification of mineralization area. (H) The levels of osteogenic marker genes were analysed by rt‐PCR. Data are shown as mean ± SD. *p < 0.05, **p < 0.01, ***p < 0.001, ****p < 0.0001

The MTT test showed that, after being pre‐incubated with NGR1 ranging from 10 to 50 μM, cell viability reduced by H_2_O_2_ was effectively recovered under the condition of 25 μM (Figure 1A). TUNEL staining and the TUNEL positive cell counting (Figure 1B, C) indicated a 40% decrease of apoptosis by treatment with NGR1. To evaluate the osteoprotective effect of NGR1, MC3T3‐E1 cells were cultured in OM. ALP, as a biomarker of osteoblast function, was observed decreased in H_2_O_2_ group and recovered 21% by supplementing NGR1 in ALP staining and ALP activity test (Figure 1D, E). The osteogenic capability of mineralization examined by ARS was also significantly rescued by NGR1 (Figure 1F, G). Furthermore, expression levels of typical osteogenic marker genes (ALP, Osteocalcin (OCN), Collagen I (COL I) and Runt‐related transcription factor 2 (Runx2)) in OS injured model decreased but largely recovered after NGR1 administration (Figure 1H).

### 3.2 NGR1 attenuated H_2_O_2_‐induced osteoblast mitochondrial dysfunction

**FIGURE 2:**
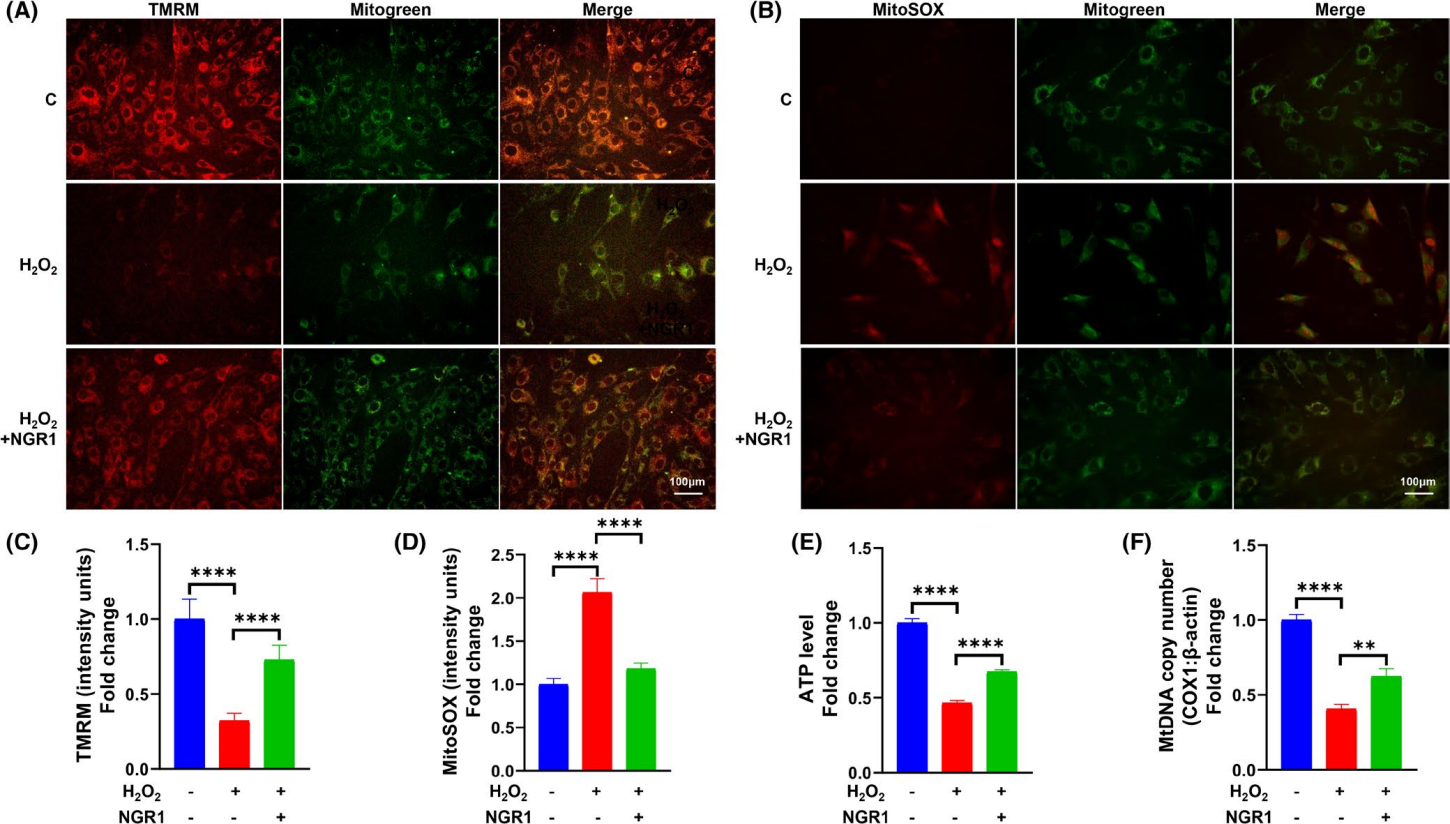
NGR1 attenuated H_2_O_2_‐induced osteoblast mitochondrial dysfunction. MC3T3‐E1 cells were incubated with 25 μM NGR1 for 24 h before treating with 0.75 mM H_2_O_2_ for 6 h. (A) Typical images and (B) quantification of MitoSOX staining; scale bar, 100 μm. (C) Typical images and (D) quantification of Tetramethylrhodamine methyl ester (TMRM) staining in the indicated groups. Mitogreen staining was performed to show the mitochondria; scale bar, 100 μm. (E) Adenosine triphosphate (ATP) levels were determined in the presence or absence of H_2_O_2_ and NGR1. (F) Mitochondrial DNA (MtDNA) copy number represented by the accompanying histograms (COX‐1: β‐actin) in each group. Data are shown as mean ± SD. **p < 0.01, ****p < 0.0001

To further confirm the role of NGR1 on mitochondrial OS and dysfunction in H_2_O_2_‐induced MC3T3‐E1 cells dysfunction, we tested MMP, mitochondrial ROS (MtROS), ATP production and MtDNA copy number. We found out that NGR1 significantly restored MMP (Figure 2A, C), ameliorated MtROS generation (Figure 2B, D), and increased ATP production as well as MtDNA copy number (Figure 2E, F). Collectively, these results indicate that NGR1 exerts efficient anti‐oxidative and mitochondria‐protective effects.

### 3.3 NGR1 blocked JNK signalling pathway activated by H_2_O_2_

**FIGURE 3:**
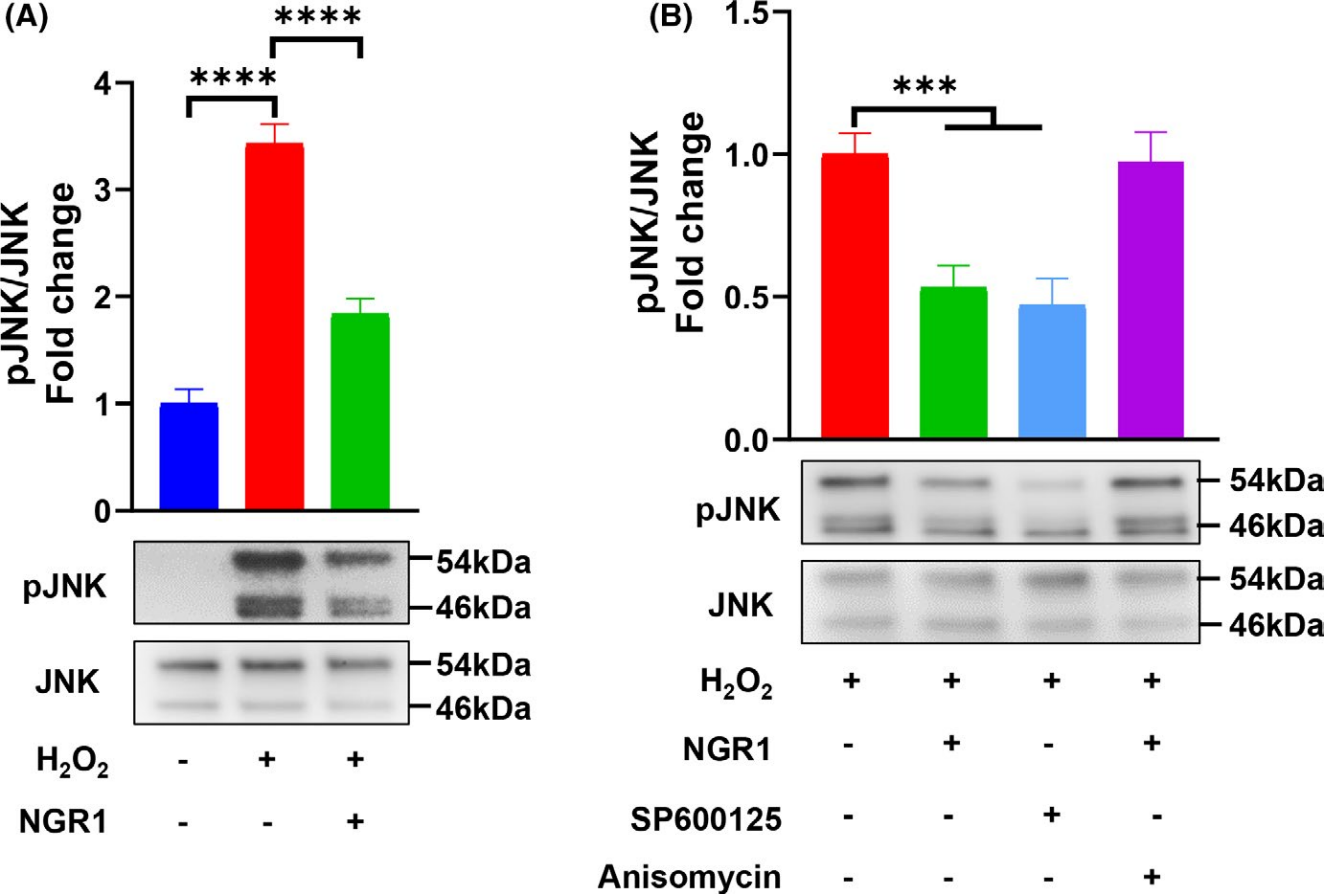
NGR1 blocked JNK signalling pathway activated by H_2_O_2_. (A) MC3T3‐E1 cells were incubated with 25 μM NGR1 for 24 h before treating with 0.75 mM H_2_O_2_ for 6 h. Representative Western blotting and the quantification of phosphorylated JNK (pJNK) relative to JNK. (B) Representative Western blotting and the quantification of pJNK relative to JNK in SP600125 or Anisomycin pretreating for 1 h groups. Data are shown as mean ± SD. ***p < 0.001, ****p < 0.0001

JNK signalling pathway activation was assessed by the expression of phosphorylated JNK compared with total JNK protein. According to the Western blot results, NGR1 significantly prevented phosphorylation of JNK by 46% (Figure 3A), and its blocking effect was similar to that of SP600125, the specific JNK inhibitor (*p* = 0.8281) (Figure 3B). However, the blocking effect of NGR1 was abolished by adding JNK agonist Anisomycin (*p* > 0.05 vs. H_2_O_2_ group) (Figure 3B). These results demonstrate that NGR1 effectively blocked H_2_O_2_‐induced JNK signalling activation.

### 3.4 NGR1 prevented H_2_O_2_‐induced osteoblast apoptosis and dysfunction by blocking JNK signalling pathway

**FIGURE 4:**
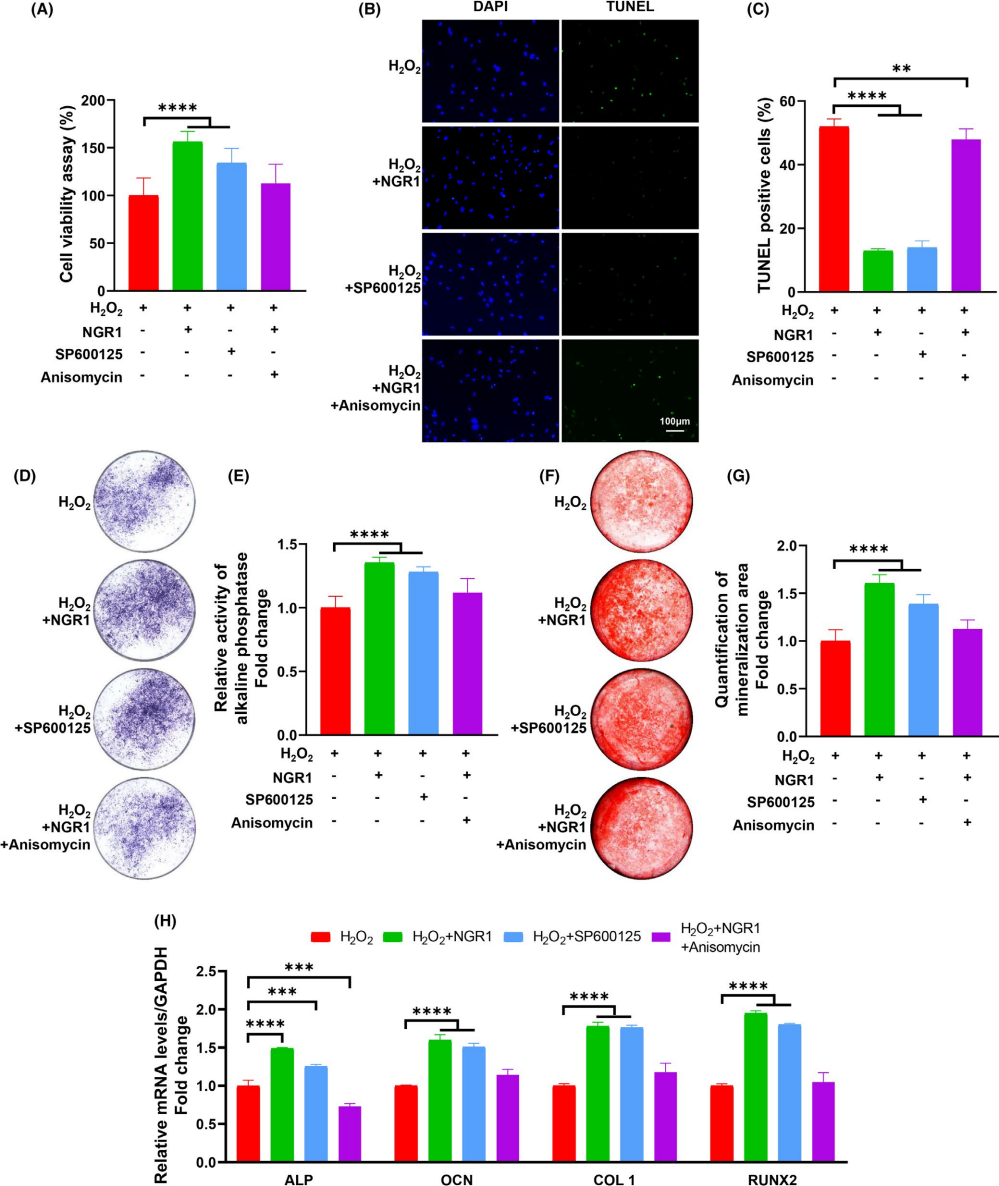
NGR1 prevented H_2_O_2_‐induced osteoblast apoptosis and dysfunction by blocking JNK signalling pathway. (A) MC3T3‐E1 cells were incubated with 25 μM NGR1 for 24 h before treating with 0.75 mM H_2_O_2_ for 6 h. SP600125 or Anisomycin was added 1 h before H_2_O_2_ treatment. Cell viability was determined by 3(4,5‐dimethylthiazol‐2‐yl)‐2,5‐diphenyltetrazolium (MTT) reduction. (B, C) Cells were stained for Transferase dUTP Nick End Labeling (TUNEL) (green). 4’, 6‐diamidino‐2‐phenylindole (DAPI) was used to stain the nuclei. Scale bars, 100 μm. (D) MC3T3‐E1 cells after indicated treatment were subjected to alkaline phosphatase (ALP) staining and (E) ALP activity test. (F) Mineralization area of MC3T3‐E1 cells after osteogenic inducing for 4 weeks was determined by alizarin red S staining. (G) The quantification of mineralization area. (H) The levels of osteogenic marker genes were analysed by rt‐PCR. Data are shown as mean ± SD. **p < 0.01, ***p < 0.001, ****p < 0.0001

To further investigate the protective effect of NGR1 and the role of JNK signalling pathway in OS‐induced osteoblast injury. JNK inhibitor SP600125 and activator Anisomycin were used in MC3T3‐E1 cells OS model. SP600125 rescued MC3T3‐E1 cells viability and prevented H_2_O_2_‐induced apoptosis, as observed in Figure 4A and TUNEL staining results (Figure 4B, C). Furthermore, the results of ALP staining (Figure 4D, E), ARS (Figure 4F, G) and osteogenic genes’ levels (Figure 4H) demonstrate that blocking JNK by SP600125 also recovered MC3T3‐E1 cells’ osteogenic ability. These data showed a stronger protective effect of NGR1 than SP600125. However, reactivating JNK by Anisomycin eliminated these benefits significantly, which confirmed that osteoblast protection from NGR1 due to its blockage of JNK signalling pathway.

### 3.5 NGR1 promoted mitochondrial function recovery by blocking JNK signalling pathway

**FIGURE 5:**
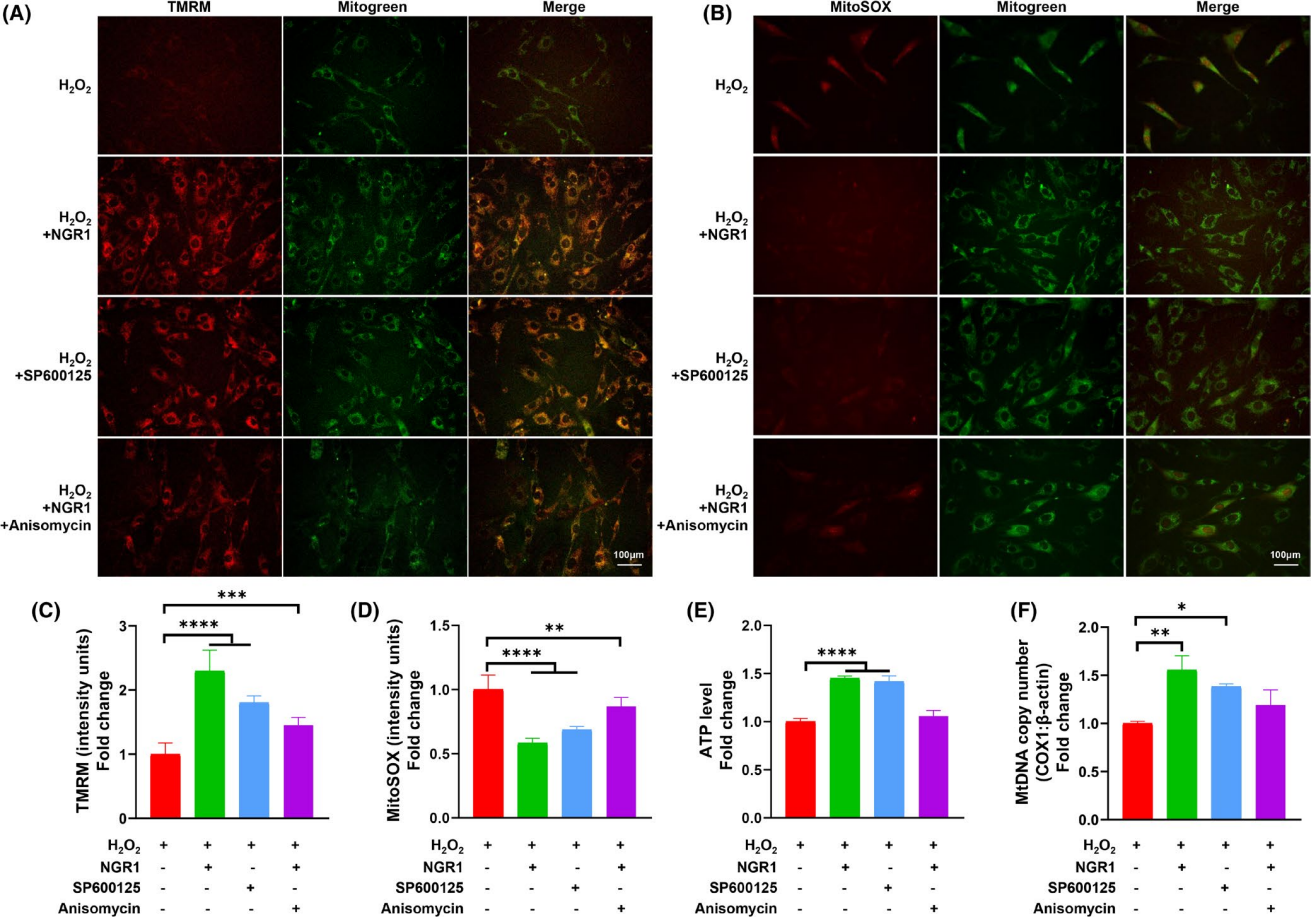
NGR1 promoted recovery of mitochondrial function by blocking JNK signalling. MC3T3‐E1 cells were incubated with 25 μM NGR1 for 24 h before treating with 0.75 mM H_2_O_2_ for 6 h. SP600125 or Anisomycin was used 1 h before H_2_O_2_ treatment. (A) Typical images and (B) quantification of MitoSOX staining; scale bar, 100 μm. (C) Typical images and (D) quantification of tetramethylrhodamine methyl ester (TMRM) staining in the indicated groups. Mitogreen staining was performed to show the mitochondria; scale bar, 100 μm. (E) Adenosine triphosphate (ATP) levels were determined in the presence or absence of H_2_O_2_ and NGR1. (F) Mitochondrial DNA (MtDNA) copy number represented by the accompanying histograms (COX‐1: β‐actin) in each group. Data are shown as mean ± SD. *p < 0.05, **p < 0.01, ***p < 0.001, ****p < 0.0001

As shown in Figure 5A–D, SP600125 restored MMP and prevented MtROS generation induced by H_2_O_2_, which represented the recovery of mitochondrial status. Blocking JNK signalling by SP600125 also rescued ATP production and MtDNA copy number (Figure 5E, F), showing the recovery of mitochondrial energy generation and mitochondrial abundance. Notably, NGR1 showed a SP600125 similar mitochondrial protective capability before adding Anisomycin compromised those benefits. Thus, JNK signalling is pivotal in mitochondrial function recovery process of NGR1.

## 4 DISCUSSION

OS‐induced mitochondrial damage and subsequent osteoblast dysfunction are revealed to contribute to the onset and development of osteoporosis.^9^ In this study, we, show for the first time that NGR1 antagonizes OS‐induced damage, restores osteoblast viability and osteogenic differentiation. At subcellular level, NGR1 significantly reduces OS‐induced MtROS and restores mitochondrial function, such as membrane potential, ATP production and MtDNA copy number. We further demonstrate a key role of JNK in OS‐induced mitochondrial damage and osteoblast dysfunction. Finally, NGR1 could block JNK activation and protect osteoblast from oxidative damages. These data indicate that NGR1 can significantly attenuate OS‐induced mitochondrial damage and restore osteogenic differentiation of osteoblast by suppressing JNK activation, thus bearing a promising potential in treating osteoporosis.

ROS consist of a number of diverse radical and non‐radical oxygen species, such as superoxide anion (O_2_^−^), hydroxyl radical (OH^−^) and H_2_O_2_, which are originally generated during normal metabolism in mitochondria following the activation of various enzymes, such as nicotinamide adenine dinucleotide phosphate (NADPH) oxidase, cyclooxygenases (COXs) and various mitochondrial oxidases.^27^, ^28^ O_2_^−^ is regarded as the ‘primary’ ROS and generates ‘secondary’ more aggressive ROS after interacting with other molecules. In both physiological conditions, such as ageing, hormonal changes^29^, ^30^, ^31^, ^32^ and pathological conditions associated with exposure to drug, radiation, exogenous or endogenous toxins and inflammatory cytokines,^33^, ^34^, ^35^ ROS override the intrinsic anti‐oxidative defence mechanism, leading to OS. ROS initially attack and cause dysfunction to mitochondria, whose damage stimulates further production of ROS, thereby exacerbating OS. OS generates cellular damage due to lipid oxidation, structural alteration of the membranes, oxidation of proteins and nucleic acids; the damage may further extend to the organs and become systemic.^36^ For example, the pathology of many metabolic disorders and degenerative diseases such as diabetes mellitus and neurological disorders are highly related to OS.^37^, ^38^, ^39^ Similarly, ROS greatly affect the generation, survival and functions of osteoblast, which is revealed to contribute to the onset and development of osteoporosis.^4^

Among ROS, H_2_O_2_ is more stable than other forms and can be added exogenously, which makes it suitable to mimic OS microenvironment.^40^ Thus, H_2_O_2_ is widely used to establish cell oxidative damage model.^41^, ^42^, ^43^ Consistent with our previous reports,^8^ we found that H_2_O_2_ administration significantly affected not only osteoblast viability and survival (Figure 1A‐C), but also osteogenic differentiation, such as ALP expression (Figure 1D, E), osteoblastogenic marker genes levels (Figure 1H) and mineralization (Figure 1F, G). OS‐induced osteoblast dysfunction is mainly initiated and exacerbated by mitochondrial dysfunctions.^44^, ^45^ Overproduction of ROS attacks mitochondrial outer and inner membrane causing mitochondrial permeability transition and inducing mitochondrial membrane depolarization accompanying with excessive MtROS production.^10^, ^46^ These damages further lead to the release of proapoptotic proteins,^47^ thereby initiating an osteoblast apoptotic program. On the other hand, mitochondria are the largest factory for ATP synthesis^48^ so as to provide energy for cell activities, such as osteoblastic differentiation.^44^ In the current study, H_2_O_2_ caused a series of severe mitochondrial dysfunctions in osteoblast, such as mitochondrial membrane depolarization (Figure 2A, C), MtROS overproduction (Figure 2B, D) and ATP level reduction (Figure 2E), which was consistent to our previous findings.^11^ In this study, we further showed that H_2_O_2_ also significantly decreased MtDNA copy number (Figure 2F)—an indicator of mitochondrial abundance and mutation,^49^ which contributed new knowledge to OS‐induced osteoblast dysfunction. Continuous efforts have been made to seek efficacious bioactive agents that can both potently restore mitochondrial functions and promote osteogenic activities.

One major resource for such bioactive agents is natural herbs‐derived small‐molecule compounds, many of which bear a potent anti‐oxidative property.^50^ Some of them have been used in the treatment of OS‐mediated systemic diseases such as neurological disorders and diabetes mellitus.^39^ In addition, the bioactive agent to treat osteoporosis should also have a potent capacity of promoting osteogenesis. One of such small‐molecule compounds is NGR1—the major bioactive agent of many Chinese medicines that applied in clinic. NGR1 has been used in clinic to manage various diseases, such as cerebrovascular and cardiovascular diseases.^13^ In animal studies, NGR1 has been proven to have various effective therapeutic functions, such as neuroprotection, anti‐diabetes, certain organ protection, bone metabolism regulation, anti‐cancer and osteoporosis, which can be largely attributed to its potent antiapoptotic, anti‐inflammatory and anti‐oxidative properties.^13^, ^51^ NGR1 has been shown to effectively protect various types of cells from oxidative damages triggered by other pathological factors, such as AGEs (advanced glycation end products),^16^ ischaemia‐reperfusion injury^52^ and oxidized low‐density lipoprotein.^53^ NGR1 can decrease ROS production and prevent protein and lipid peroxidation by improving the activities of antioxidant enzymes, such as superoxide dismutase (SOD), catalase and glutathione.^15^, ^52^, ^54^ As expected, our current study also showed that NGR1 antagonized the H_2_O_2_‐induced OS, restored cell viability and decreased apoptosis rate of osteoblast (Figure 1A–C). In the meantime, since NRG1 also bears a potent pro‐osteogenic property,^19^ we further tested its efficacy in OS microenvironment in this study. Our results showed that NGR1 could restore ALP level and activity (Figure 1D, E), extracellular mineralization (Figure 1F, G) and the levels of a series of osteoblastogenic marker genes (such as ALP, OCN, COL I and Runx2) (Figure 1H) that were suppressed by H_2_O_2_. Mechanistically, Zhang et al. show that the anti‐oxidative effects of NGR1 are mediated by a well‐known transcriptional factor Nrf2 and its subsequent HO‐1 signalling.^16^ On the other hand, the suppression of JNK is also shown to play an important role of NGR1 in protecting cells from OS damages.^55^ However, the exact role of JNK remains unveiled.

JNK is one of the three well‐characterized MAPK pathways, which transduce extracellular signals and regulate various cell activities, such as cell proliferation, migration, differentiation and apoptosis.^56^ Serving as a critical stress‐responsive pathway, JNK is directly involved in the mitochondrial‐dependent pathway of apoptosis.^57^, ^58^ In addition, activated JNK also leads to the impairment of mitochondrial function and the inactivation of JNK rescues the biogenesis of mitochondria, recovers mitochondrial respiration and ATP synthesis in many kinds of cells.^23^, ^59^, ^60^ These functional characteristics of JNK inspired us to hypothesize that NGR1 maintained mitochondrial function so as to prevent OS‐induced osteoblast dysfunction through suppressing JNK activation. Consistent with previous studies,^17^, ^61^ our data show that NGR1 reduces MtROS and restores MMP, ATP synthesis and MtDNA copy number in OS *in vitro* microenvironment (Figure 2), which confirms that NGR1 can restore mitochondrial function. We further show that NGR1 effectively blocks the JNK signalling (Figure 3). The critical role of JNK in mitochondrial functions was corroborated by the findings that JNK inhibitor (SP600125) mimicked while JNK agonist (Anisomycin) blocked the protective effects of NGR1 on osteoblast dysfunction and mitochondrial dysfunction against OS (Figures 4 and 5). With all these findings, we demonstrated that the blockage of JNK signalling pathway contributed to NGR1’s mitochondria and osteoblast protection in OS conditions.

In fact, it has been previously suggested that NGR1 regulates JNK signalling through various pathways. Firstly, JNK is an important signalling protein downstream of Protein kinase B (Akt), NGR1 treatment can increase the expression of phospho‐Akt and reduce the activity of the JNK signalling pathway in rat brain hypoxic‐ischaemic injury model.^55^ In addition, phospho‐inositol requiring enzyme‐1(IRE1) activates the JNK pathway, while NGR1 inhibits activation of phospho‐IRE1 in primary cortical neuron.^25^ In lipopolysaccharide‐triggered human lung fibroblast injury model, TAK1, also known as mitogen‐activated protein kinase kinase kinase 7 (MAP3K7), activates the downstream JNK pathway, which can be downregulated by NGR1.^62^ MicroRNA like miR‐132 is also involved in the NGR1‐induced JNK signalling pathway blockage in different kinds of cells.^53^, ^63^, ^64^ Further studies should be performed to investigate the molecular mechanisms involved in the axis of NGR1‐JNK‐mitochondrial function.

One limitation in this study is the adoption of a mouse osteoblast cell line. Studies with primary human mesenchymal stem cells need to be performed before extrapolating the current findings to clinical situations. Furthermore, in our future study, we will perform a well‐designed *in vivo* study to illustrate the therapeutic effect of NGR1 on osteoporosis.

## 5 CONCLUSION

NGR1 significantly attenuates OS‐induced mitochondrial damage and restores osteogenic differentiation of osteoblast through the blockage of JNK signalling pathway. This finding revealed the molecular biological mechanisms for the preventing effect of NGR1 on OS‐induced osteoblast dysfunction, which lays a theoretic foundation for the further application of NGR1 for the prevention and treatment of osteoporosis.

## CONFLICTS OF INTEREST

The authors confirm that there are no conflicts of interest.

## AUTHORS’ CONTRIBUTIONS

**Xumin Li**: Data curation (equal); Funding acquisition (equal); Writing‐original draft (equal). **Haiyan Lin**: Data curation (equal); Writing‐original draft (equal); and Investigation (equal). **Xiaorong Zhang, Qihao Yu and Yinghui Ji**: Data curation (equal). **Richard T. Jaspers**: Supervision (equal); Validation (equal); and Writing‐review & editing (equal). **Tim Forouzanfar**: Supervision (equal); Formal analysis (equal); and Project administration (equal). **Dongyun Wang**: Formal analysis (equal); and Funding acquisition (equal). **Shengbin Huang**: Supervision (equal); Project administration (equal); Investigation (equal); and Conceptualization (equal). **Gang Wu**: Supervision (equal); Project administration (equal); Funding acquisition (equal); Writing‐review & editing (equal); and Conceptualization (equal).

## Data Availability

The data sets generated for this study are available on request to the corresponding author.
